# Melatonin as an immunomodulator in children with Down syndrome

**DOI:** 10.1038/s41390-021-01611-6

**Published:** 2021-08-16

**Authors:** Dean Huggard, Lynne Kelly, Amy Worrall, Eleanor Gallagher, Lida Fallah, Lucas Lu Yoo, Fiona McGrane, Niamh Lagan, Edna Roche, Joanne Balfe, Derek G. Doherty, Eleanor J. Molloy

**Affiliations:** 1 Paediatrics, Trinity College, The University of Dublin & Trinity Research in Childhood Centre (TRiCC), Dublin, Ireland; 2 Trinity Translational Medicine Institute (TTMI), St James Hospital, Trinity College Dublin, Dublin, Ireland; 3 Paediatrics, Children’s health Ireland (CHI) at Tallaght, Dublin, Ireland; 4 National Children’s Research Centre, Dublin, Crumlin, Ireland; 5 Neonatology, Coombe Women and Infants University Hospital, Dublin, Ireland; 6 Neonatology, CHI at Crumlin, Dublin, Ireland

## Abstract

**Background:**

Down syndrome (DS) is a disorder characterised by marked immune dysfunction, increased mortality from sepsis, chronic inflammation, increased oxidative stress, sleep disturbance and possibly abnormal endogenous melatonin levels. Melatonin has a myriad of immune functions, and we hypothesised that this therapeutic agent could modulate the innate immune system in this cohort.

**Methods:**

We investigated neutrophil and monocyte function (CD11b, TLR4 expression by flow cytometry), genes involved in TLR signalling (MyD88, IRAK4, TRIF), the inflammasome (NLRP3, IL-1β), and circadian rhythm (BMAL, CLOCK, CRY) by qPCR, and inflammatory cytokines (IL-2, IL-6, IL-8, IL-18, IL-1β, TNF-α, IFN-γ, IL-10, IL-1ra, VEGF, Epo, GM-CSF) by enzyme-linked immunosorbent assay (ELISA) following immunomodulation with LPS endotoxin and melatonin. 47 children with DS and 23 age- and sex-matched controls were recruited.

**Results:**

We demonstrated that melatonin has several significant effects by reducing CD11b and TLR4 expression, attenuating TLR signalling, genes involved in the inflammasome and has the potential to reduce LPS-induced inflammatory responses.

**Conclusions:**

Immunomodulatory effects of melatonin were found in both paediatric cohorts with more marked effects in the children with DS. Melatonin mediates immune response through a wide array of mechanisms and this immunomodulator may buffer the inflammatory response by regulating pro and anti-inflammatory signalling.

**Impact:**

- We highlight that melatonin mediates its immune response through a wide array of mechanisms, its effects appear to be dose dependant and children with Down syndrome may be more receptive to treatment with it.
- Immunomodulatory effects of melatonin were demonstrated with marked effects in the children with Down syndrome with a reduction of MyD88, IL-1ß and NLRP3 expression in whole-blood samples.
- Melatonin is a proposed anti-inflammatory agent with a well-established safety profile, that has the potential for mitigation of pro- and anti-inflammatory cytokines in paediatric Down syndrome cohorts, though further clinical trials are warranted.

## Introduction

Down syndrome (DS) is the most prevalent chromosomal abnormality worldwide, affects 1 in 700 births in the United States, and is due to extra genetic material from chromosome 21^1^. It is associated with several co-morbidities including, low IQ, cognitive delay, congenital heart disease, gastrointestinal anomalies, obstructive sleep apnoea, and acute leukaemia^2^. The immune system is also dysregulated in DS with many abnormalities described; decreased T and B lymphocyte counts^3,4^, suboptimal antibody responses to vaccination^5–7^, altered serum cytokines^8,9^, abnormal neutrophil chemotaxis^10^, and higher levels of autoimmunity and interferon-related signalling proteins^11^.

From a clinical perspective these immune deficits contribute to a vulnerable population as children with DS are more prone to infections, admission to hospital with respiratory tract infections (RTIs), and more often require longer hospital stays^12,13^. Furthermore, in the setting of sepsis, a 30% increased mortality has been reported in children with DS^14^. This may partly be due to the high circulating levels of pro-inflammatory cytokines in this cohort^8,15^, leading to uncontrolled inflammatory cascades, systemic inflammatory response syndrome (SIRS), sepsis and ultimately poorer outcomes. Other reasons for the immune dysfunction are thought to due directly due to.

Another consequence of an altered immune system is that chronic inflammation and autoimmunity; arthropathy, coeliac and thyroid disease, are found more commonly^16–18^. Periodontal disease is due to persistent inflammation in the oral cavity and is also more prevalent in DS, this and other chronic peripheral infections have been implicated in the possible pathogenesis of neurodegenerative changes or Alzheimer’s disease, which occurs with regularity and at an earlier onset in DS^19,20^. Trisomy 21 also causes overexpression of the enzyme superoxide dismutase, consequently, an excess of free radicals and increased oxidative stress is hypothesised to occur in this cohort, resulting in early-onset AD and cataracts^21^.

Melatonin (*N*-acetyl-5-methoxytryptamine) is an endogenous multifunctional hormone, which is secreted in the pineal gland and other organs by animals, including humans. This versatile molecule has also been preserved in more primitive species such as bacteria, and phylogenetics has tracked its origins to be over 3 billion years old^22^. The effects of melatonin on regulating circadian rhythm are well described^23,24^. However it is now known that there are several important immunomodulatory effects of this molecule, especially in relation to benefits as an adjuvant in treating sepsis, chronic inflammation, and as an abrogator of neurodegeneration^25–27^, all of which occur more frequently in DS. Melatonin has been shown to have strong antioxidant effects, antiapoptotic activity through inhibition of caspase 3 cleavage, and anti-inflammatory actions via inflammasome deactivation^28^.

Melatonin has a myriad of immune functions and we aimed to evaluate if its effects extend further by examining neutrophil and monocyte function, genes involved in TLR signalling, the inflammasome, and pro and anti-inflammatory cytokines following immunomodulation with lipopolysaccharide (LPS; endotoxin) and melatonin. We hypothesised that melatonin could have potentially significantly mediate aspects of the innate immune system in children with DS. This is especially pertinent as DS is characterised by marked immune dysfunction, increased mortality from sepsis, chronic inflammation, prone to oxidative stress, sleep disturbance and possibly abnormal endogenous melatonin levels^29,30^.

## Materials and methods

### Study population

The Ethics committees of Children’s Health Ireland at Tallaght and Crumlin, Dublin, Ireland both approved the study. Both verbal and written informed consent was sought from all families and participants prior to recruitment. Two patient groups were enrolled: (a) Children with Down syndrome <16 years of age attending the Down syndrome clinic, in Tallaght and Crumlin (b) Controls (age-matched): patients for routine phlebotomy or day ward procedures. In this instance, blood sampling took place at the start of gas induction general anaesthetic. Neither cohort had any recent evidence of infection or fever.

### Experimental design

We used similar experimental methods (as described below), as per our previous studies Huggard et al.^31–33^. Sodium citrate anti-coagulated tubes were used for collecting and transporting blood (1–3 mL) samples for in vitro experiments. These were analysed within 2 h of sample acquisition. Whole blood was incubated at 37 °C for 1 h with lipopolysaccharide (LPS; E.coli 0111:B4: SIGMA Life Science, Wicklow, Ireland) 10 ng/mL, melatonin at 42 μM, and then both together.

### Quantification of cell-surface antigen expression

Blood samples were incubated with a dead cell stain (100 μL; (Fixable Viability Dye eFlour 506, Invitrogen, California)), diluted to working concentration in phosphate-buffered saline (PBS). The following fluorochrome-labelled monoclonal antibodies (mAb) were added to each sample (2.5 μL per tube): CD14-PerCP, CD15-PECy7, CD16-FITC, CD66b-Pacific Blue and TLR4-APC (BioLegend®, California) and PE labelled CD11b (BD Biosciences, Oxford, UK; 10 μL per tube). PBA buffer (PBS containing 1% bovine serum albumin and 0.02% sodium azide) was used to make up the antibody cocktail. Samples were incubated in the dark for 15 min. Next 1 mL of FACS lysing solution (BD Biosciences, Oxford, UK) was added to each tube, the samples were then incubated for 15 min in the dark. Cells were pelleted by centrifugation at 450×*g* for 7 min at room temperature, washed twice with PBA buffer and fixed in 300 μL of 1% paraformaldehyde. The final cell pellet was resuspended in 100 μL PBA buffer and analysed on a BD FACS Canto II flow cytometer^31,32^.

The expression of CD11b and TLR4 antigens on the surface of neutrophils and monocytes was evaluated by flow cytometry on the BD FACS Canto II cytometer on neutrophils and monocytes from 20 children with DS and age and sex-matched controls. Neutrophils were delineated based on SSC-A and CD66b^+^ positivity as previously described^34^, monocytes were defined based on SSC-A, CD66b- and subsets based on relative CD14^+^CD16^+^ populations; classical (CD14^+^/CD16^−^), intermediate (CD14^+^/CD16^+^), non-classical (CD14dim/CD16^+^). A minimum of 10,000 events was collated and relative expression of CD11b and TLR4 was expressed as mean channel fluorescence (MFI), and analysed using FloJo software (Oregon). Every sample was processed and analysed by the same researcher (DH) thereby reducing variability in results^31,32^.

### Inflammasome and circadian gene analysis

Following incubation of samples with the immunomodulators, 1 mL of Trizol (ThermoFisher) solution was added to 0.3 mL of whole blood. The samples were incubated for 5 min at room temperature followed by the addition of chloroform. Following lysis, the aqueous phase was used to isolate RNA, as per the manufacturer’s instructions (Invitrogen, US). RNA purity and concentration were determined by using the NanoDrop ND-100 Spectrophotometer and analysed using ND-1000 Ver.3.1.2 software. Total RNA, 1 µg, was reverse transcribed to single-stranded cDNA using the High-Capacity cDNA Archive Kit (Applied Biosystems) following the manufacturer’s protocol and stored at −80 °C until use. The settings for amplification were 10 min at 25 °C and 120 min at 37 °C and 5 min at 85 °C then hold at 4 °C. The evaluation of gene expression was performed by Taqman® RT-PCR. Commercially available TaqMan® primer and probe combinations were used to detect expression of the following TLR signalling genes, MyD88 (NM 001172567.1), TRIF (NM_182919.3) and IRAK4 (NM_001114182.2), the following inflammasome genes, NLRP3 (NM_001079821.2), IL-1β (NM_000576.2), ASC (NM_ 013258.4), and the following circadian rhythm genes, BMAL (NM_001030272.2), Clock (NM_001267843.1), CRY (NM_004075.4), REV-ERB-α (NM_021724.4). The endogenous control selected was GAPDH. All samples were assayed in triplicate. Thermal cycling conditions were as follows: 2 min at 50 °C, 10 min at 95 °C, and for 40 cycles, 24 s at 95 °C and 1 min at 60 °C, using the 7900HT Fast Real-Time *PCR* System. Relative quantification (RQ) values were calculated using the 2^−ΔΔCt^ method^32^.

### Cytokine analysis

The following cytokines; tumour necrosis factor-alpha (TNFα), interleukin 1β (IL-1β), interleukin 6 (IL-6), interleukin 8 (IL-8), interferon-gamma (IFN-γ), interleukin 18 (IL-18), vascular endothelial growth factor (VEGF), erythropoietin (Epo), interleukin 1 receptor antagonist (IL-1ra), and interleukin 10 (IL-10) were analysed using a custom-made MSD®MULTI-SPOT assay plate from Mesoscale (MSD Diagnostics, Rockville, MD). Extracted peripheral blood serum, as described above, was transferred to a 96 well MSD plate and these cytokines were assessed as per the manufacturer’s instructions. The plate was then analysed on the SECTOR Imager and validated (Meso Scale Discovery, Rockville, MD; www.meso-scale.com). The limits of detection for the individual assays were within expected ranges^32^.

### Statistics

Statistical analysis was performed using unpaired *t*-tests to compare mean results between two independent cohorts, with Benjamini-Hochberg correction to adjust for false discovery rate controlling for multiple comparisons. Significance was defined as p < 0.05. Results shown are expressed as mean ± standard error of the mean (SEM) unless otherwise stated. Data were analysed with FloJo software (Oregon) and GraphPad Prism.

## Results

### Patient characteristics

The participants with DS (*n* = 47) had a mean age (mean ± SD) of 5.1 ± 4.3 years (y), of which 42.6% were female, and the controls (*n* = 23) were 7.7 ± 4.2 y old of which 52.2% were female^31^. In the DS cohort, 74.4% (*n* = 35) had a diagnosis of CHD, with 25.5% (*n* = 12) requiring corrective surgery. The controls were healthy children without DS and no major co-morbidities.

### Effects of melatonin on cytokine responses in DS vs controls

**Fig. 1:**
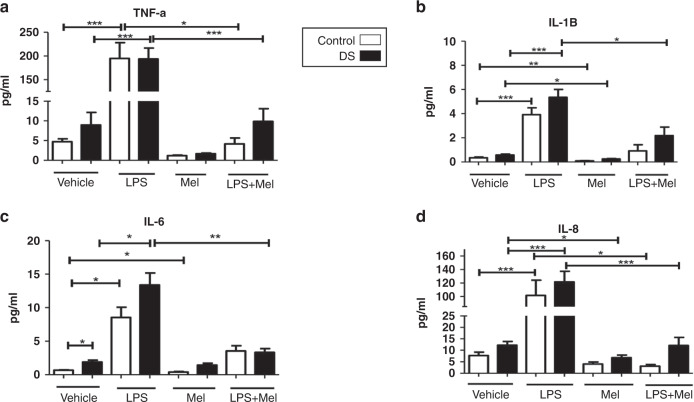
Cytokine levels of TNF-α, IL-1β, IL-6, IL-8 in response to lipopolysaccharide (LPS) and melatonin in children with Down syndrome and controls. Cytokine levels in the plasma of TNF-α (**a**), IL-1β (**b**), IL-6 (**c**), IL-8 (**d**) at baseline and in response to lipopolysaccharide (LPS) and melatonin in in vitro samples from children with DS (*n* = 47) and controls (*n* = 23). Values expressed as pg/mL. *p < 0.05, **p < 0.01, ***p < 0.001.

**Fig. 2:**
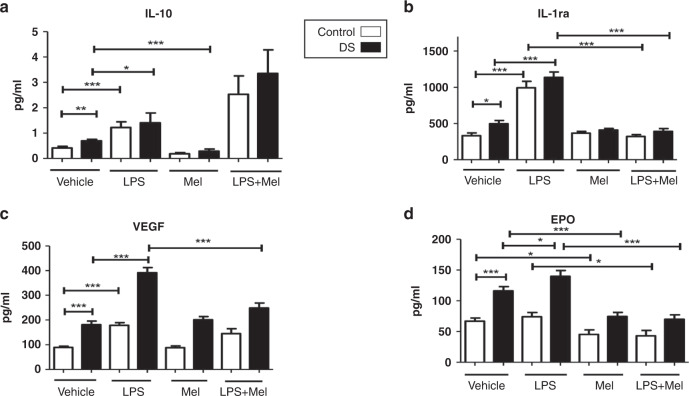
Cytokine levels of IL-10, IL-1ra, VEGF, Epo in response to lipopolysaccharide (LPS) and melatonin from children with Down syndrome and controls. Cytokine levels in the plasma of IL-10 (**a**), IL-1ra (**b**), VEGF (**c**), Epo (**d**) at baseline and in response to lipopolysaccharide (LPS) and melatonin in in vitro samples from children with DS (*n* = 47) and controls (*n* = 23). Values expressed as pg/mL. *p < 0.05, **p < 0.01, ***p < 0.001.

**Fig. 3:**
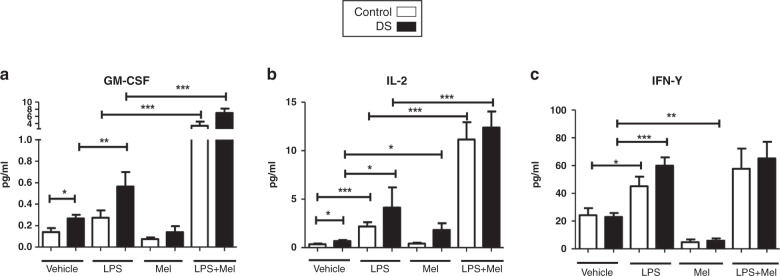
GM-CSF, IL-2, IFN-y in response to lipopolysaccharide (LPS) and melatonin in children with Down syndrome and controls. Cytokine levels in the plasma of GM-CSF (**a**), IL-2 (**b**), IFN-y (**c**) at baseline and in response to lipopolysaccharide (LPS) and melatonin in in vitro samples from children with DS (*n* = 47) and controls (*n* = 23). Values expressed as pg/mL. *p < 0.05, **p < 0.01, ***p < 0.001.

Compared with baseline levels, treatment with melatonin resulted in significant decreases in IL-1β and Epo in both cohorts (*p* < 0.05; Figs. 1b, 2d). IL-8, IL-10, and IFN-γ were also reduced compared with baseline after melatonin incubation but only in children with DS (Figs. 1d, 2b and 3c). Melatonin caused an increase in IL-2 in children with DS, and a decrease in IL-6 in controls vs baseline (Figs. 1c and  3b). The following cytokines did not change significantly in either group following melatonin incubation: IL-18, TNFα, VEGF, GM-CSF, IL-1ra (Figs. 1–3).

### Effects of melatonin on LPS-induced cytokine responses including melatonin

Comparing cytokine levels after LPS treatment with samples treated with LPS plus melatonin there were significant reductions in TNF-α, IL-8, IL-18, Epo and IL-1ra in both cohorts (*p* < 0.05) (Fig. 1a, d and 2d, b). In children with DS, there were significant decreases in IL-1β, IL-6 and VEGF comparing LPS treated samples and those treated with LPS and melatonin, this was not replicated for controls (Figs. 1b, c and 2c). For IL-2 and GM-CSF, treatment with both LPS and melatonin resulted in significant increases in these cytokines in both cohorts, and although not quite reaching statistical significance the same trend was observed for IFN-γ and IL-10. (Figs. 2a and 3a–c).

### Effects of melatonin on CD11b and TLR4

**Fig. 4:**
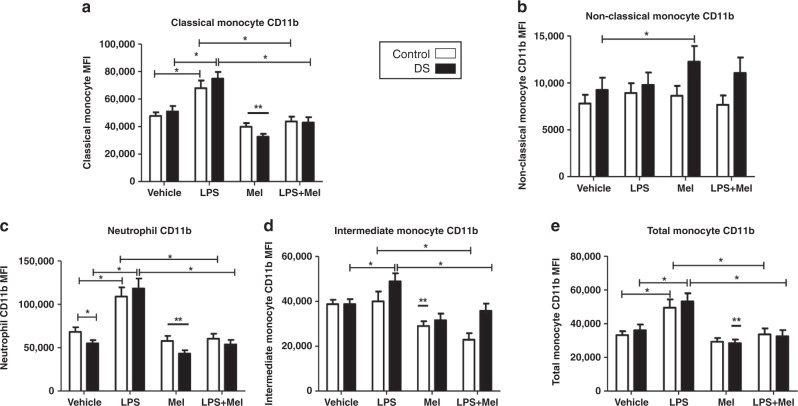
Neutrophil and monocyte CD11b expression in response to lipopolysaccharide (LPS) and melatonin in children with Down syndrome (DS) and controls. Values expressed as mean channel fluorescence (MFI). *p < 0.05, **p < 0.05 vs vehicle in the corresponding cohort. **a** Neutrophil CD11b (DS *n* = 23; controls *n* = 16). **b** Total monocyte CD11b (DS *n* = 19; controls *n* = 21). **c** Classical monocyte CD11b (DS *n* = 19; controls *n* = 21). **d** Intermediate monocyte CD11b (DS *n* = 18; controls *n* = 20). **e** Non-classical monocyte (DS *n* = 19; controls *n* = 21).

Compared with baseline expression melatonin caused significant decreases in CD11b on neutrophils and classical monocytes in both cohorts (*p* < 0.05) (Fig. 4a, c). There was a significant reduction in total monocyte CD11b in children with DS, and a significant reduction in intermediate monocyte CD11b in controls, when compared to baseline levels (Fig. 4b, d). A paradoxical increase in CD11b on non-classical monocytes was observed in the DS cohort (Fig. 4e).

**Fig. 5:**
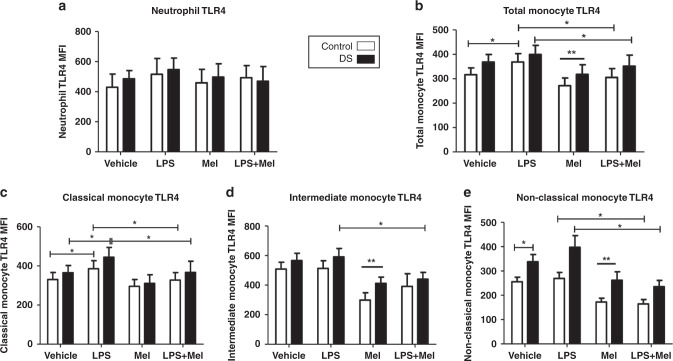
Neutrophil and monocyte Toll-like receptor (TLR4) expression in response to lipopolysaccharide (LPS) and melatonin in children with Down syndrome (DS) and controls. Values expressed as mean channel fluorescence (MFI). *p < 0.05, **p < 0.05 vs vehicle in the corresponding cohort. **a** Neutrophil TLR4 (DS *n* = 19; controls *n* = 10). **b** Total monocyte TLR4 (DS *n* = 22; controls *n* = 15). **c** Classical monocyte TLR4 (DS *n* = 16; controls *n* = 15). **d** Intermediate monocyte TLR4 (DS *n* = 15; controls n = 14). **e** Non-classical monocyte TLR4 (DS *n* = 16; controls *n* = 20).

When comparing samples treated with LPS alone with those incubated with LPS and melatonin, there were significant decreases in CD11b expression in all cell sub-types (neutrophils, total, classical, intermediate monocytes) in both cohorts, except for the non-classical monocyte (*p* < 0.05) (Fig. 4a–d). There was no difference observed in the latter. Melatonin treatment resulted in significant TLR4 reductions on total, intermediate and non-classical monocytes in both cohorts when compared to baseline (*p* < 0.05) (Fig. 5b, d, e). There was no effect of melatonin on neutrophils or classical monocytes (Fig. 5a, c).

Evaluating TLR4 expression between samples treated with LPS and those treated with LPS plus melatonin revealed significant falls in the receptor on total, classical and non-classical monocytes in both groups (*p* < 0.05) (Fig. 5b, c, e). On the intermediate monocytes, there was a significant reduction in TLR4 in the DS cohort only. No difference was observed for neutrophils in either group (Fig. 5a, d). Data pertaining to vehicle and LPS samples only is as previously published^31^.

### Melatonin and the inflammasome

**Fig. 6:**
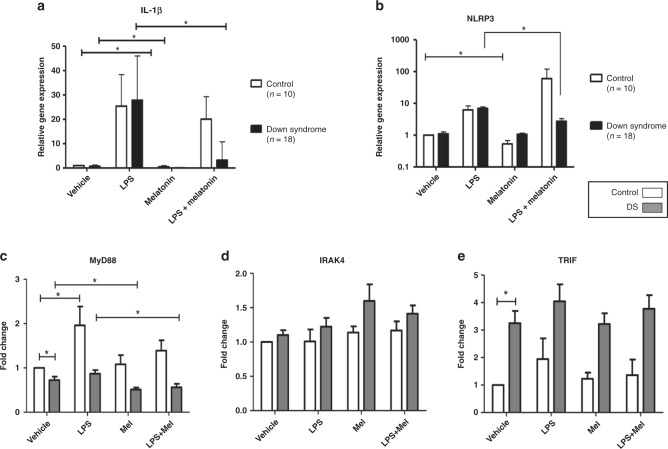
IL-1β, NLRP3, IRAK4, MyD88 and TRIF children with DS compared to controls. Fold-change expression of IL-1β (**a**), and NLRP3 (**b**), in whole-blood samples from children with DS (*n* = 18) compared to control samples (*n* = 10), and of MyD88 (**c**), IRAK4 (**d)**, TRIF (**e**), in children with DS (*n* = 10) and controls (*n* = 10) at baseline and following treatment with LPS and melatonin. Statistical significance is *p < 0.05.

Compared with baseline expression melatonin caused a significant reduction in IL-1β in both cohorts (DS *p* < 0.0001; control *p* < 0.0001) (Fig. 6a). NLRP3 expression was decreased with melatonin in the controls but not in children with DS (*p* = 0.002) (Fig. 6b). There were no differences in ASC expression following melatonin treatment in either group. When samples treated with LPS alone were compared to those incubated with LPS and melatonin there were significant decreases in IL-1β (*p* < 0.0001), and NLRP3 (*p* = 0.0001) in children with DS but not controls (Fig. 6a, b). There was no change in ASC following the above comparisons.

### Melatonin and the TLR pathway, circadian rhythm

Melatonin had the effect of reducing MyD88 expression compared to baseline in children with DS but not in controls (*p* = 0.02) (Fig. 6c). When compared to baseline melatonin did not result in any changes in gene expression for IRAK4 or TRIF in either cohort (Fig. 6d, e). Comparing samples treated with LPS vs those treated with LPS and melatonin, the addition of melatonin resulted in a significant decrease in MyD88 expression in children with DS (*p* = 0.017), but not in controls (Fig. 6c). There were no changes in IRAK4 or TRIF following the above treatments (Fig. 6d, e). Data pertaining to vehicle and LPS samples only is as previously published^32^. There were no significant differences at baseline or comparing LPS to LPS plus melatonin in the expression of BMAL, CLOCK, CRY, REV-ERBα in both cohorts (Supplementary Fig. S1).

## Discussion

We have demonstrated that melatonin has several significant effects on innate immunity, innate immune cells, receptors, and TLR pathways, and has the potential to reduce endotoxin-induced inflammatory responses. Immunomodulatory effects of melatonin were found in both paediatric cohorts; however, it appears there may be more marked effects in the children with DS in relation to cytokine release, TLR signalling and genes involved in the inflammasome. To our knowledge, this has not been carried out previously in this cohort or in healthy paediatric controls.

Melatonin is produced in the pineal gland at the base of the brain and exerts its effects through several mechanisms. Firstly, melatonin can mediate its actions via direct contact and interaction with molecules; antioxidant activity through chelation of reactive nitrogen and reactive species^35^. As hormone melatonin also functions through binding cell membrane receptors; MT1 and MT2 (located on almost all peripheral and CNS tissues), which once activated stimulate phospholipase A & C, ultimately reducing cGMP and cAMP^36^. Furthermore, melatonin appears to have activity on receptors located in the nucleus also; retinoid Z receptors (RZR) and retinoid orphan receptors (ROR)^37^. However, this special hormone’s varied array of effects leads us to further consider other cells, receptors and pathways that may also be affected.

In our study, melatonin lowered CD11b on neutrophils and monocytes and TLR4 on monocytes compared to baseline and following LPS plus melatonin treatment in both cohorts. CD11b is a key receptor involved in the activation and diapedesis of immune cells promoting the inflammatory response^38^, TLR4 is crucial in recognising LPS endotoxin, stimulating the innate response, and implicated in septic shock^39^. This demonstrates the ability of melatonin to reduce cell activation, migration and abrogate TLR4 signal activation at the cell surface, respectively. This could have implications for improving outcomes in acute inflammation and sepsis or in chronic disease as both CD11b and TLR4 are associated with these processes^40,41^.

The analysis of the influence of melatonin on cytokines yielded perhaps the most significant results. In both cohorts comparing LPS with LPS plus melatonin treated samples there were significant reductions in TNF-α, IL-8, IL-18, IL-1ra, Epo, in both groups. However, a more pronounced melatonin response was observed in the DS cohort for certain mediators. In response to melatonin IL-8, IL-10 and IFN-γ were reduced compared to controls. After LPS and melatonin levels were compared to LPS, there were significant decreases in IL-1β, IL-6, and VEGF only in the children with DS. It is well described that melatonin can abrogate pro-inflammatory cytokine release. One mechanism of action is by impairing NF-κB transcriptional activity, reducing pro-inflammatory cytokine (IL-1β, TNF-α, IFN-γ) release^42,43^. It may be that this cohort may respond more effectively to melatonin immunomodulation and should be included in further experiments to elucidate this fully.

Sepsis is defined as a life-threatening organ dysfunction caused by a dysregulated host response to infection and it is associated with significant morbidity, mortality and sizeable economic costs^44^. The metabolic pathways affected by sepsis lead to imbalances in cytokine production, oxidative stress and mitochondrial function^45,46^. Targeted effects of melatonin on different cytokines in response to LPS sepsis in mice demonstrated that mice treated with the immunomodulator had much higher survival rates (90% vs 20%), and those pro-inflammatory cytokines such as TNF-α, IFN-γ, and IL-12 were reduced, the anti-inflammatory IL-10 was significantly raised^47^. Shang et al.^48^ and Xu et al.^49^, also reported melatonin to result in significant increases in IL-10 in this context. This may lead to a more balanced cytokine response to infection and improve outcomes in sepsis. In our study, there was a trend towards significant rises in IL-10 in both cohorts, and we did observe increases in IL-2 and GM-CSF for both groups. There is evidence that melatonin can augment IL-2 levels in monocytes ex vivo and that it may play a role in regulating CD4^+^ lymphocyte activity^50^.

Lipopolysaccharide (LPS) endotoxaemia is the commonest cause of sepsis^51^, and the biochemical alterations translate to cell death and apoptosis leading to septic shock and multiorgan dysfunction. Rodent research has now shown that these clinical effects of endotoxaemia can be improved by the administration of melatonin in conjunction with standard treatments^52,53^. In sepsis, the mechanism of action of melatonin has been studied Hu et al.^54^ reported improved mitochondrial function and inhibition of oxidative and nitrosative stress. Indeed, the robust antioxidant or free radical scavenging activity of melatonin is highlighted in several studies^55,56^. In animal models of LPS-induced mastitis, melatonin has been shown to decrease the inflammatory response by reducing oxidative stress and pro-inflammatory cytokines such as IL-1β, IL-6, GM-CSF^57,58^. Novel research has demonstrated that in a murine model of polymicrobial sepsis, neutrophils and macrophages exhibit specific melatonin receptors and melatonin can augment neutrophil antimicrobial actions by promoting neutrophil extracellular traps (NET), and improve survival^59^.

Much of the literature (above) focuses on animal or ex vivo experimentation on melatonin to assess its role as an immunomodulator, however, there are some paediatric studies published which examined its use in patients with sepsis. Gitto et al.^60^, reported improved outcomes and survival in neonates with suspected sepsis (*n* = 10) given melatonin vs controls (*n* = 10). Improved mitochondrial function and restoration of ATP production was cited by the authors as one of the key reasons behind the improvement in clinical status in the treatment arm. A further study evaluating melatonin in a neonatal population (Rx *n* = 25, control *n* = 25) with sepsis found that there were better clinical and laboratory outcomes in those who received melatonin^61^. Naveen Kumar et al. evaluated melatonin in both mouse and adult neutrophils, in vivo and ex vivo, and demonstrated that melatonin protects neutrophils from oxidative stress-induced apoptosis by reducing ROS generation; but also, in contrast, it restores neutrophil functions like phagocytosis, degranulation, and NETosis in GSH and GR activity-deficient neutrophils by regulating ROS levels. They suggest that melatonin might be a potential auxiliary therapy for immune dysregulation^62^. Although these studies highlight the potential utility of melatonin as adjuvant therapy in managing sepsis, these were non-randomised, non-blinded trials and the need for the establishment of large RCTs to examine melatonin further is needed.

In our study, MyD88 gene expression was reduced compared to baseline with melatonin and following LPS and melatonin treatment in the DS cohort only. This suggests melatonin can mediate its effects on TLR signalling in DS. TLR receptors generate signals via two main pathways based on whether the adaptor protein MyD88 is used: MyD88-dependent or MyD88-independent pathways. All TLRs apart from TLR3 are MyD88-dependent and ultimately result in NF-kB and TNF production. The latter pathway signals through another adaptor, TRIF, inducing IRF3 causing IFN-β to be released^63^. We previously found a decrease in MyD88 and an increase in TRIF at baseline in children with DS vs controls^32^. This potentially contributes to the increased susceptibility to recurrent infection in children with DS and suggests anomalous TLR signalling in DS can have deleterious consequences due to dysregulated inflammatory cytokine release.

The potential effects of melatonin on the TLR signalling pathways have not been elucidated in detail. However, Kang et al.^64^ evaluated this by examining protein expression of MyD88, NF-kB, TRIF, IRF3 and IFN-β following liver ischaemia and reperfusion injury in mice. The authors report significant reductions in all of the above TLR signalling proteins, and attenuation of IL-6 and TNF-α, suggesting that melatonin plays a key role as an immunomodulator in TLR pathways. Further insights into the mechanism by which melatonin may act on TLR4 and its pathway are provided by Fu et al.^65^, who describes how TLR4 and MyD88 expression were reduced through inhibition of TLR4 and MyD88 binding, and because of restriction of TLR/MD2/CD14 complex formation by melatonin. This is supported by Tamtaji et al.^66^, who commented on a similar mechanism and importance of melatonin as a TLR inhibitor. As we have demonstrated an excess of TLR4 on non-classical monocytes in children with DS^31^, melatonin may prove to be of therapeutic benefit in this cohort, with further research required.

In evaluating melatonin and genes involved in inflammasome activation we discovered that on comparing LPS and LPS plus melatonin there were significant decreases in IL-1β and NLRP3 expression in the children with DS but not in controls. The inflammasome is part of the innate immune system and is a multiprotein complex that ultimately generates pro-inflammatory cytokines IL-1β and IL-18^67^. The NLRP3 inflammasome was a source of interest given its association with chronic inflammation and autoimmunity^68^, more prevalent in DS, and the reported greater circulating IL-1β levels in DS^8,15^. There appears to be a potential role for melatonin in inhibiting inflammasome activation; Liu et al.^69^ in a murine model of sepsis reported that the following induction by LPS and melatonin, NLRP3 inflammasome activation was reduced. Similarly, Rahim et al.^70^ reported that melatonin counteracts the NF-kB/NLRP3 connection in response to LPS, and postulate that this agent may have a role in attenuating the inflammatory response by this mechanism. Given that a greater response to melatonin was seen in the DS cohort, and the increased susceptibility to autoinflammation in this population also, specific targeting of the inflammasome could prove beneficial.

Our evidence suggests that there is a preferential response to melatonin in the DS cohort in relation to certain cytokine responses, TLR signalling and genes involved in inflammasome activation. One potential contributor is that the numbers in the DS group were lower than the controls for the cytokine experiments. Another may be that as a population, those with DS may exhibit altered endogenous melatonin levels. Of the few studies examining this, there is conflicting evidence; Uberos et al.^30^ looked at serum melatonin levels in children with DS (*n* = 15) and controls (*n* = 15), and reported lower levels in the DS group, ((pointing out a possible inherent vulnerability to oxidative stress from free radical accumulation)). Contrary to this Reiter et al.^21^ examined the chief metabolite of melatonin, 6-hydroxymelatonin sulphate, in urine over 24 h (DS *n* = 12). The absolute levels and variations in this melatonin metabolite were the same as controls. As we have outlined, sleep disruption is common in children with DS, which may potentially disturb their circadian rhythm causing anomalous melatonin levels and immune responses. Effects of disruption to circadian clocks and the immune response is discussed by Labrecque et al.^71^, who reports that multiple cellular functions are disrupted: cellular migration, proliferation in response to pathogens, phagocytosis and cytolysis. In a Down syndrome mouse model evaluating sleep and circadian rhythm, it was found that although the mice did exhibit sleep interruptions similar to patients with DS, their circadian rhythm remained unaffected^72^. Fernandez et al.^73^ performed a cross-sectional study examining sleep and circadian rhythm periods in young children with DS (*n* = 66, age range 5–67 months) and controls (*n* = 43, age-matched normal development). They observed that children with DS had more sleep fragmentation the circadian rhythms were robust, and the authors conclude that any resultant cognitive delays are not exacerbated by alterations in circadian rhythms. This has been borne out in our work examining genes involved in regulating circadian rhythm by qPCR. We found that there were no significant differences in key genes BMAL, CLOCK, CRY and REV-ERBα, in in vitro samples from children with DS (*n* = 10) and controls (*n* = 10) at baseline or after immunomodulation with LPS and melatonin. Future studies might look at subgroup analyses of children with DS who have congenital heart disease, or obstructive sleep apnoea, with sleep questionnaires, and correlations with immune markers. The Espinosa group have investigated the underlying deficits in children with DS, in particular, those arising directly from the triplication of several immune-related genes on the q arm of Hsa21, including four of the six interferon receptors (IFNAR1, IFNAR2, IFNGR2, and IL10RB) and several interferon-related signalling proteins operating within the innate immune system (MX1, MX2, CCT8)^11^ and these too are worthy of future studies in our DS cohorts. Similarly, a circadian signalling timeline series to capture full clock gene expression changes in DS and controls would ideally be completed in future research.

Overall melatonin mediates its immune response through a wide array of mechanisms, its effects appear to be dose dependant and children with DS may be more receptive to treatment with it. Furthermore, this immunomodulator may play a key role in buffering the inflammatory response by regulating pro and anti-inflammatory signalling depending on the context. A beneficial safety profile also makes this agent the subject of many further clinical trials^74^, which are much needed to fully elucidate the actions of this pleiotropic hormone.
